# Melatonin alleviates intervertebral disc degeneration by disrupting the IL-1β/NF-κB-NLRP3 inflammasome positive feedback loop

**DOI:** 10.1038/s41413-020-0087-2

**Published:** 2020-02-18

**Authors:** Fan Chen, Guowei Jiang, Hui Liu, Zemin Li, Yuxin Pei, Hua Wang, Hehai Pan, Haowen Cui, Jun Long, Jianru Wang, Zhaomin Zheng

**Affiliations:** 1 Department of Spine Surgery, The First Affiliated Hospital of Sun Yat-sen University, Guangzhou, 510080 China; 2 Department of Pediatric Intensive Care Unit, The First Affiliated Hospital of Sun Yat-sen University, Guangzhou, 510080 China; 3 Pain Research Center, Sun Yat-Sen University, Guangzhou, 510080 China

**Keywords:** Neurophysiology, Metabolism

## Abstract

The inflammatory response is induced by the overexpression of inflammatory cytokines, mainly interleukin (IL)-1β, and is one of the main causes of intervertebral disc degeneration (IVDD). NLR pyrin domain containing 3 (NLRP3) inflammasome activation is an important source of IL-1β. As an anti-inflammatory neuroendocrine hormone, melatonin plays various roles in different pathophysiological conditions. However, its roles in IVDD are still not well understood and require more examination. First, we demonstrated that melatonin delayed the progression of IVDD and relieved IVDD-related low back pain in a rat needle puncture IVDD model; moreover, NLRP3 inflammasome activation (NLRP3, p20, and IL-1β levels) was significantly upregulated in severely degenerated human discs and a rat IVDD model. Subsequently, an IL-1β/NF-κB-NLRP3 inflammasome activation positive feedback loop was found in nucleus pulposus (NP) cells that were treated with IL-1β. In these cells, expression of NLRP3 and p20 was significantly increased, NF-κB signaling was involved in this regulation, and mitochondrial reactive oxygen species (mtROS) production increased. Furthermore, we found that melatonin disrupted the IL-1β/NF-κB-NLRP3 inflammasome activation positive feedback loop in vitro and in vivo. Melatonin treatment decreased NLRP3, p20, and IL-1β levels by inhibiting NF-κB signaling and downregulating mtROS production. Finally, we showed that melatonin mediated the disruption of the positive feedback loop of IL-1β in vivo. In this study, we showed for the first time that IL-1β promotes its own expression by upregulating NLRP3 inflammasome activation. Furthermore, melatonin disrupts the IL-1β positive feedback loop and may be a potential therapeutic agent for IVDD.

## Introduction

Low back pain (LBP), one of the most common health problems, is a leading cause of disability worldwide and results in an enormous global burden to public health and the social economy, and ~84% of people experience LBP some point in their lifetime.^1–6^ LBP is a multifactorial disease,^7^ and the disorder is strongly associated with intervertebral disc degeneration (IVDD), which is characterized by a homeostatic imbalance between anabolism and catabolism, including extracellular matrix (ECM) degradation^8,9^ or nucleus pulposus (NP) cell survival.^10–12^ The intervertebral disc (IVD) is a special organ that consists of an outer fibrocartilaginous annulus fibrosus (AF) and an inner gel-like NP.^13,14^ Inflammatory responses, which are induced by inflammatory cytokine overexpression, are a primary and important cause of IVDD. Recent studies have demonstrated that inflammatory cytokines, including tumor necrosis factor (TNF)-α and interleukin (IL)-1β, are strongly correlated with ECM degradation or NP cell survival.^15,16^ Therefore, a more profound understanding of the molecular mechanisms underlying inflammatory cytokine secretion might provide new therapeutic targets for IVDD.

The NLR pyrin domain containing 3 (NLRP3) inflammasome, a primary and crucial source of the highly inflammatory cytokines IL-1β and IL-18, is a canonical multimeric inflammasome complex that is composed of the adaptor apoptosis-associated speck-like protein containing a CARD (ASC) and the effector pro-caspase-1.^17,18^ When exposed to exogenous or endogenous stimuli, the NLRP3 inflammasome becomes activated and drives caspase-1 activation, which results in the cleavage and maturation of IL-1β and IL-18.^19^ Dysregulated NLRP3 inflammasome activation is involved in diverse diseases, including neurodegenerative diseases,^20,21^ osteoarthritis,^22^ cancer,^23,24^ and inflammatory diseases.^25,26^ Few studies have investigated the relationship between NLRP3 inflammasome activation and IVDD. However, numerous studies have confirmed that IL-1β is an important cause of IVDD,^16,27^ and so we hypothesized that the NLRP3 inflammasome activation/IL-1β inflammatory response axis may play an important role in IVDD progression and that eliminating stimuli that activate the NLRP3 inflammasome may alleviate this progression.

Melatonin (N-acetyl-5-methoxytryptamine), which is synthesized by the pineal gland and many other organs, is a neuroendocrine hormone^28,29^ that is involved in a wide range of physiological functions, including anti-inflammatory,^30,31^ antidegenerative,^32,33^ antioxidant,^34,35^ immunomodulatory,^36,37^ circadian rhythm regulation,^38^ and cancer prevention activities.^39–42^ Notably, recent studies have demonstrated that melatonin attenuates the inflammatory response by inhibiting NLRP3 inflammasome activation during the progression of atherosclerosis^43^ and brain,^44^ liver^45^ and lung diseases.^46^ Moreover, melatonin plays crucial roles in the IVDD process, including regulating NP cell proliferation, remodeling the ECM,^47^ protecting vertebral endplate chondrocytes against apoptosis and calcification,^48^ and preventing oxidative stress-induced NP cell apoptosis,^49^ indicating a strong correlation between melatonin and IVDD. Although these published studies have indicated that melatonin participates in the IVDD process by regulating NLRP3 inflammasome activation in NP cells, this hypothesis has not been experimentally examined.

Based on these observations, the objective of this study was to explore the molecular mechanisms underlying a novel activation model of the NLRP3 inflammasome in IVDD. We also aimed to investigate whether exogenous melatonin administration prevents IVDD by regulating NLRP3 inflammasome activation in vitro and in vivo.

## Results

### Melatonin ameliorates the progression of IVDD and LBP in vivo

**Fig. 1:**
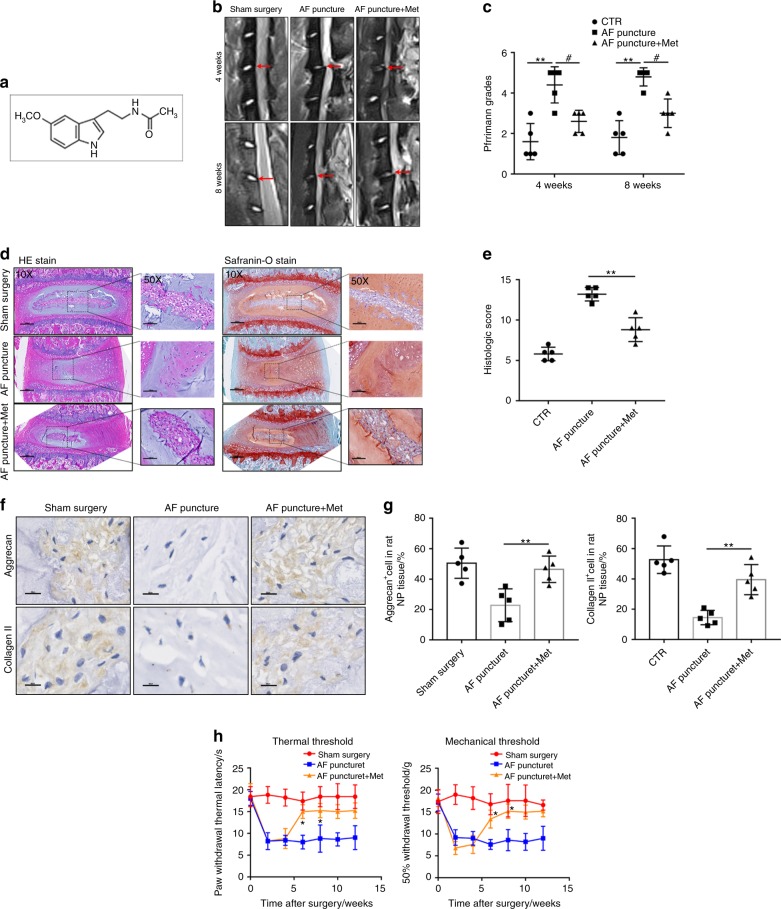
Melatonin delays the progression of IVDD in vivo. **a**, **b** MRI images and Pfirrmann grade analysis of the rat models at 4 or 8 weeks after the operation. **c**, **d** H&E staining, Safranin-O staining and histologic score analysis of the rat models at 8 weeks after the operation (original magnification ×10, ×50; scale bar = 500 µm, 200 µm). **e**, **f** IHC staining and quantitative analysis of Aggrecan and Collagen II in IVDD rats treated with melatonin (original magnification ×400; scale bar = 50 µm). **g** Behavioral study of the rat models at different time points after the operation. CTR, Control. *n* = 5, *P < 0.05; **P < 0.01; ^#^*P* < 0.05. The data are shown as the means ± SD

First, we established a rat IVDD model to determine whether melatonin exerts a protective effect during the progression of IVDD in vivo. Melatonin was intraperitoneally injected into rats with AF puncture-induced IVDD. MRI images were obtained 4 or 8 weeks post operation, and the IVDs from the rats treated with melatonin displayed a significantly higher signal intensity than those without melatonin treatment (Fig. 1b, c). Histologically, hematoxylin and eosin (H&E) and Safranin-O staining showed that the amounts of gelatinous NP tissue and the disc height in the melatonin-treated rats were larger than those in the AF puncture rats, while the histologic score of the AF puncture rats was higher than that of the melatonin-treated rats (Fig. 1d, e). IHC results showed that the expression of Aggrecan and Collagen II was decreased significantly in the AF puncture group, and the levels of Aggrecan and Collagen II were increased in rats that were treated with melatonin (Fig. 1f, g). Subsequently, the behavioral study results showed that melatonin significantly decreased mechanical hyperalgesia and thermal hyperalgesia compared to those of the AF puncture group (Fig. 1h). Therefore, these results indicated that melatonin alleviates the progression of IVDD and LBP.

### Melatonin alleviates IVDD by inhibiting NLRP3 inflammasome priming and activation in vivo

**Fig. 2:**
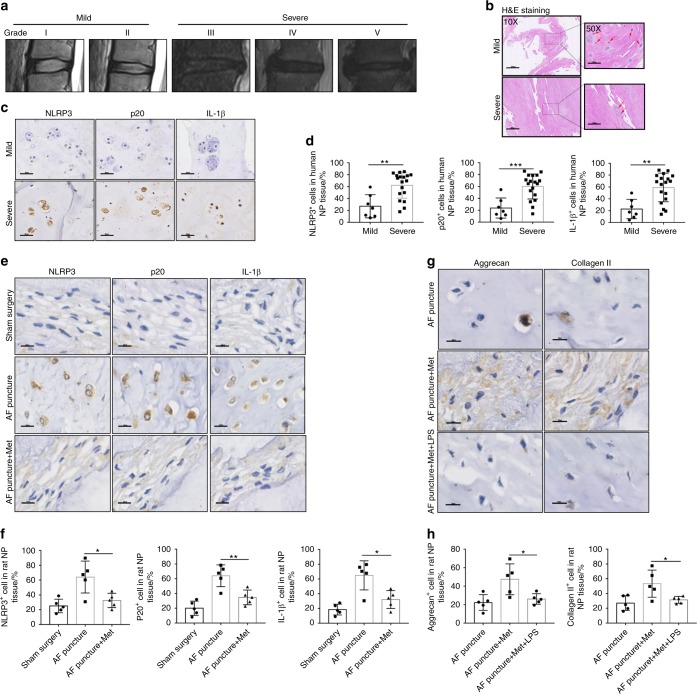
Melatonin alleviates IVDD by inhibiting NLRP3 inflammasome priming and activation in vivo. **a** MRI images of human discs. **b** H&E staining of human discs (original magnification ×10, ×50; scale bar = 500 µm, 100 µm). **c**, **d** IHC staining and quantitative analysis of NLRP3, P20, and IL-1β in human discs (original magnification ×400; scale bar = 50 µm). **e**, **f** IHC staining and quantitative analysis of NLRP3, P20, and IL-1β in IVDD rats treated with melatonin (original magnification ×400, scale bar = 50 µm). **g**, **h** IHC staining and quantitative analysis of Aggrecan and Collagen II in IVDD rats treated with melatonin or melatonin plus LPS (original magnification ×400; scale bar = 50 µm). *n* = 5, *P < 0.05; **P < 0.01; ***P < 0.001. The data are shown as the means ± SD

Next, we further investigated the correlation between NLRP3 inflammasome priming and activation and IVDD in vivo. First, human discs with different grades of degeneration are shown as representative MRI images in Fig. 2a (grades I–V). H&E staining showed that the number of NP cells was decreased in severely degenerated human discs (Fig. 2b). Moreover, the expression of NLRP3, p20, and IL-1β was increased in severely degenerated human discs compared with that in mildly degenerative human discs, as shown by IHC staining (Fig. 2c, d). Furthermore, the expression of NLRP3, p20, and IL-1β was increased in the IVDD rat model group compared with that of the control group, and melatonin administration reduced NLRP3, p20, and IL-1β expression in the IVDD rat model (Fig. 2e, f). Then, to examine the mechanism by which melatonin alleviates the progression of IVDD, we examined Aggrecan and Collagen II levels in the rat model. The expression of Aggrecan and Collagen II was significantly decreased in the AF puncture with melatonin and LPS groups (Fig. 2g, h). These results demonstrated that NLRP3 inflammasome priming and activation were involved in the process of IVDD and that melatonin alleviated IVDD by inhibiting NLRP3 inflammasome priming and activation in vivo.

### Melatonin suppresses NLRP3 inflammasome priming and activation in vitro

**Fig. 3:**
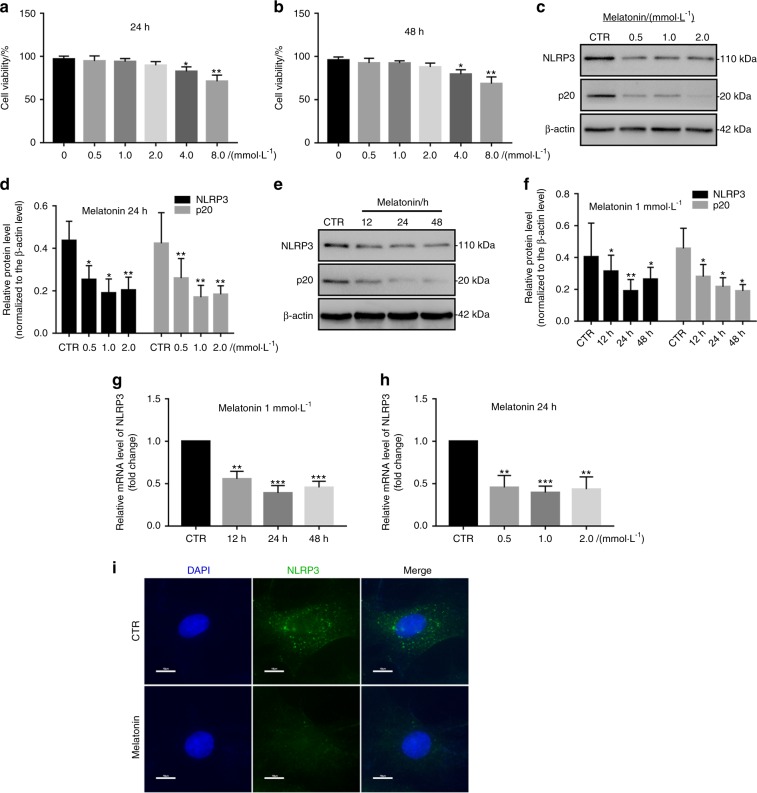
Melatonin inhibits NLRP3 inflammasome priming and activation in NP cells. **a** The chemical structure of melatonin. **b**, **c** NP cell viability after exposure to 0–8 mmol·L^−1^ NP for 24 h or 48 h. **d**–**g** Western blot and quantitative analysis of NLRP3 and P20 in NP cells treated with different doses of melatonin or for different lengths of time. **h**, **i** RT-qPCR analysis of NLRP3 and P20 in NP cells treated with different doses of melatonin or at different time points. **j** IF analysis of NLRP3 in NP cells treated with melatonin (original magnification ×1 000; scale bar = 10 µm). CTR, Control. *P < 0.05; **P < 0.01. The data are shown as the means ± SD

First, we elucidated the effect by which melatonin affects NP cell viability. Compared with the cytotoxicity in the control group, the groups treated with melatonin at concentrations below 4 mM for either 24 or 48 h did not show any obvious cytotoxic effects (Fig. 3a, b). Subsequently, while investigating the effect of melatonin on NLRP3 inflammasome priming and activation in NP cells, we found that NLRP3 and p20 expression were decreased in NP cells treated with different melatonin doses, with the lowest measured levels occurring at a dose of 1 mM (Fig. 3c, d). Furthermore, the expression levels of NLRP3 and p20 started to decrease in NP cells treated with melatonin for different lengths of time and were significantly reduced at 24 h (Fig. 3e, f). The RT-qPCR results for NLRP3 were in agreement with the western blot analysis results (Fig. 3g, h). Furthermore, IF analysis also showed that melatonin suppressed NLRP3 inflammasome activation in NP cells (Fig. 3i). These results showed that melatonin suppresses NLRP3 inflammasome activation in vitro.

### IL-1β induces NLRP3 inflammasome priming and activation in vitro

**Fig. 4:**
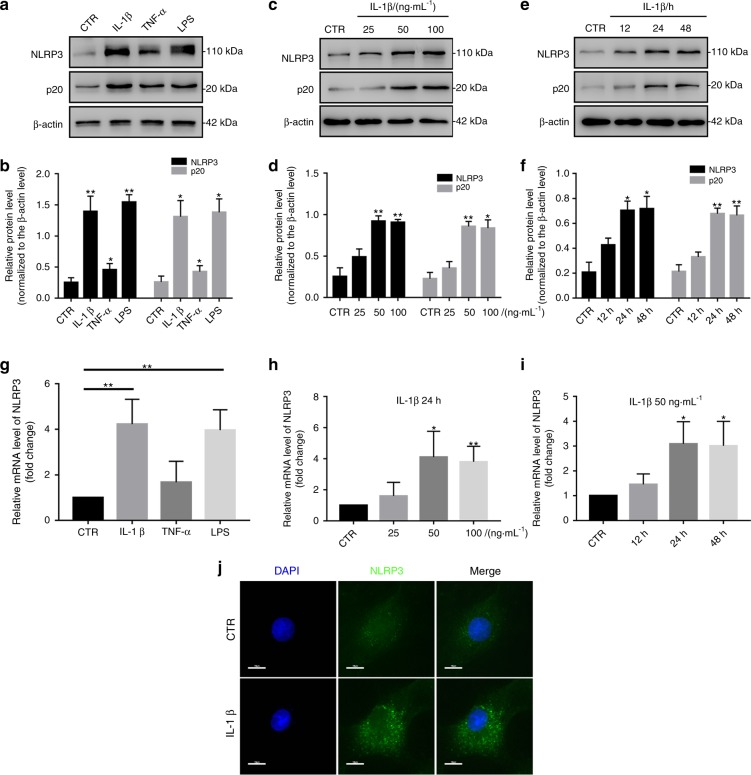
IL-1β enhances NLRP3 inflammasome priming and activation in vitro. **a**, **b** Western blot and quantitative analysis of NLRP3 and P20 in NP cells treated with IL-1β, TNF-α or LPS. **c**–**f** Western blot and quantitative analysis of NLRP3 and P20 in NP cells treated with different doses of IL-1β or at different time points. **g**–**i** RT-qPCR analysis of NLRP3 in NP cells treated with IL-1β, TNF-α or LPS, different doses of IL-1β or at different time points. **j** IF analysis of NLRP3 in NP cells treated with IL-1β (original magnification ×1 000; scale bar = 10 µm). CTR, Control. *P < 0.05; **P < 0.01. The data are shown as the means ± SD

Then, we investigated whether NLRP3 inflammasome priming and activation were induced in an IVDD cell model. As described in previous studies, IL-1β and TNF-α are classical cytokines that are used to establish an IVDD cell model. IL-1β is also produced by NLRP3 inflammasome activation. LPS is a classical stimulator of the NLRP3 inflammasome. Therefore, we selected IL-1β, TNF-α, and LPS to stimulate NP cells. First, NLRP3 and p20 expression was significantly increased in NP cells treated with IL-1β or LPS but only slightly increased in NP cells treated with TNF-α (Fig. 4a, b). Furthermore, the levels of NLRP3 and p20 gradually increased in NP cells treated with different doses of IL-1β and peaked at a dose of 50 ng·mL^−1^ (Fig. 4c, d). After IL-1β treatment for different lengths of time, the expression of NLRP3 and p20 started to increase at 12 h and exhibited an obvious increase at 24 h (Fig. 4e, f). The RT-qPCR results for NLRP3 expression were in agreement with the western blot analysis results (Fig. 4g–i). In addition, IF staining also showed that IL-1β treatment increased NLRP3 expression in NP cells (Fig. 4j). These results suggest that IL-1β enhances NLRP3 inflammasome activation in vitro.

### IL-1β upregulates NLRP3 inflammasome priming and activation by increasing NF-κB signaling and mtROS production in vitro

**Fig. 5:**
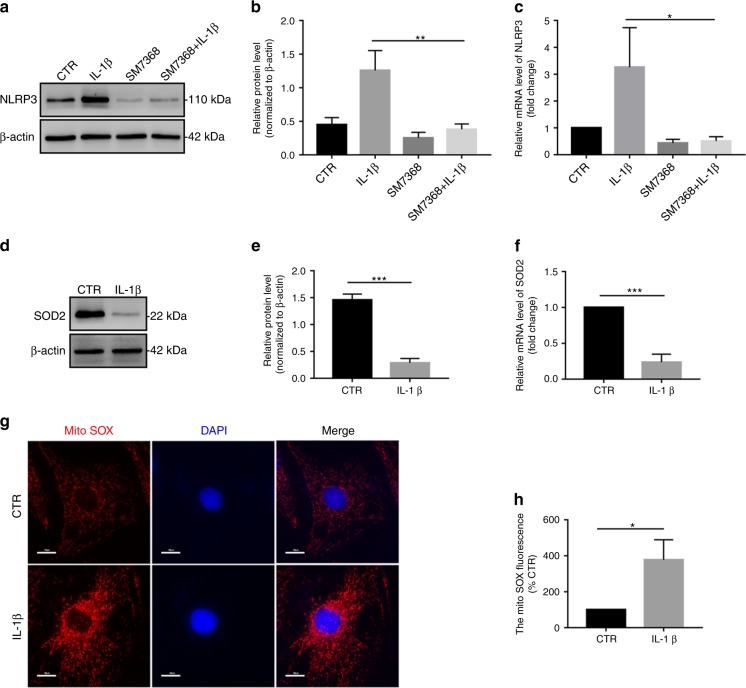
The IL-1β/NF-κB-NLRP3 inflammasome positive feedback loop is established in vitro. **a**, **b** Western blot and quantitative analysis of NLRP3 in NP cells treated with IL-1β, SM7368, or IL-1β plus SM7368. **c** RT-qPCR analysis of NLRP3 in NP cells treated with IL-1β, SM7368, or IL-1β plus SM7368. **d**–**f** Western blot and RT-qPCR analysis of SOD2 in NP cells treated with IL-1β. **g**, **h** MitoSOX red staining and quantitative analysis of NP cells treated with IL-1β (original magnification ×1 000; scale bar = 10 µm). CTR, Control. *P < 0.05; **P < 0.01; ***P < 0.001. The data are shown as the means ± SD

To examine the molecular mechanism by which IL-1β upregulates NLRP3 inflammasome priming and activation, we detected NF-κB signaling and mitochondrial reactive oxygen species (mtROS) production in NP cells. Previous studies have demonstrated that IL-1β is strongly associated with the NF-κB signaling pathway.^50,51^ In this study, we found that IL-1β activated the NF-κB signaling pathway in NP cells (Fig. S1). Subsequently, to test the role of NF-κB signaling in NLRP3 inflammasome priming in IL-1β-treated NP cells, a silencing experiment was performed. Pretreatment with SM7368 (a specific inhibitor of the NF-κB signaling pathway) or SM7368 plus IL-1β significantly decreased NLRP3 levels in NP cells compared with those of untreated NP cells (Fig. 5a–c).

As previously described, mtROS production plays an important role in NLRP3 inflammasome activation.^52,53^ Therefore, we examined whether mtROS production was involved in NLRP3 inflammasome activation in IL-1β-treated NP cells. We first detected that SOD2 expression was significantly decreased in the IL-1β treatment group compared with that of the control group, as measured by western blot and RT-qPCR analyses (Fig. 5d–e). MitoSOX Red staining indicated that mtROS production was significantly upregulated in IL-1β-treated NP cells (Fig. 5g, h). Taken together, these results confirmed that an IL-1β/NF-κB-NLRP3 inflammasome positive feedback loop is involved in the process of IVDD.

### Melatonin disrupts the IL-1β-NLRP3 inflammasome positive feedback loop in vitro

**Fig. 6:**
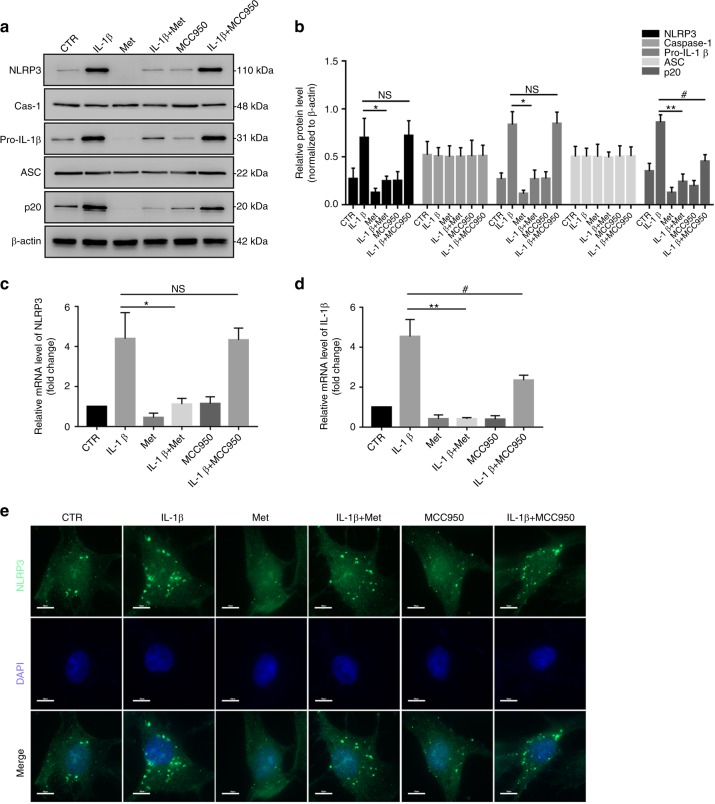
Melatonin disrupts the IL-1β-NLRP3 inflammasome positive feedback loop in vitro. **a**, **b** Western blot and quantitative analysis of NLRP3, Caspase-1, pro-IL-1β, ASC, and P20 in NP cells treated with IL-1β, melatonin, IL-1β plus melatonin, MCC950, and MCC950 plus IL-1β. **c**, **d** RT-qPCR analysis of NLRP3 and pro-IL-1β in NP cells treated with IL-1β, melatonin, IL-1β plus melatonin, MCC950, and MCC950 plus IL-1β. **e** IF analysis of NLRP3 in NP cells with different treatments (original magnification ×1 000; scale bar = 10 µm). CTR, Control; Met, melatonin; Met + IL-1β, melatonin plus IL-1β; NS, no statistical significance. *P < 0.05; **P < 0.01; ^#^*P* < 0.05. The data are shown as the means ± SD

The preceding results indicated that melatonin suppresses NLRP3 inflammasome priming and activation in NP cells. Therefore, we determined whether melatonin reduces the inflammatory response by disrupting the IL-1β-NLRP3 inflammasome positive feedback loop in NP cells. First, western blot analysis showed that melatonin obviously attenuated the IL-1β-induced increase in NLRP3, pro-IL-1β, and p20, while MCC950 (a specific inhibitor of NLRP3 inflammasome activation) did not change the IL-1β-induced upregulation of NLRP3 and pro-IL-1β. In addition, a considerable decrease in p20 levels was observed in NP cells treated with melatonin plus IL-1β (Fig. 6a, b). The RT-qPCR and western blot analysis results of NLRP3 and pro-IL-1β are shown in Fig. 6c, d. In addition, IF staining also confirmed that melatonin significantly attenuated IL-1β-induced NLRP3 inflammasome activation (Fig. 6e). These data confirmed that melatonin effectively disrupted the IL-1β-NLRP3 inflammasome positive feedback loop in vitro.

### Melatonin disrupts the IL-1β-NLRP3 inflammasome positive feedback loop by downregulating NF-κB signaling and mtROS production

**Fig. 7:**
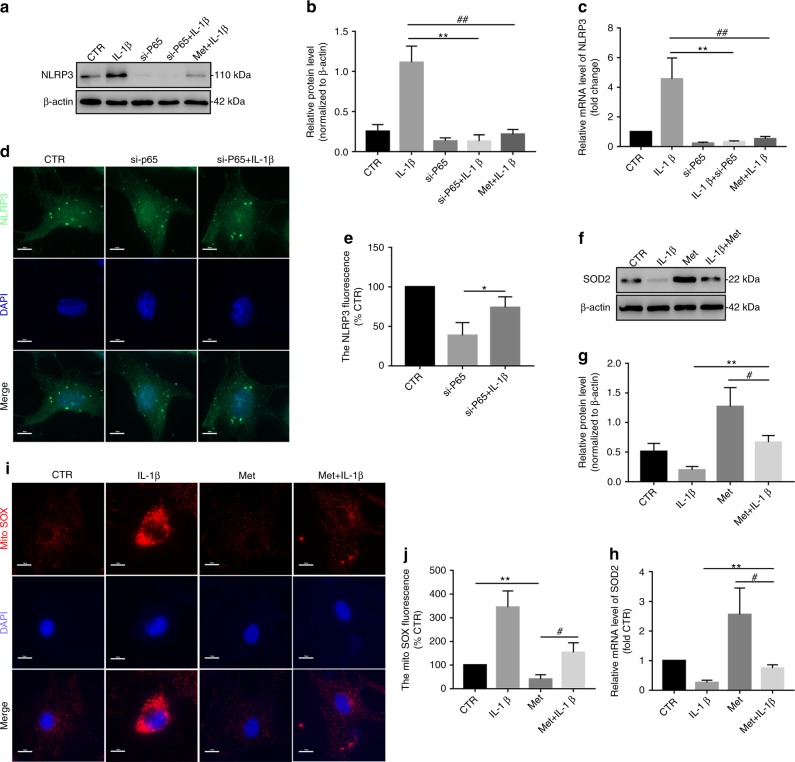
Melatonin disrupts the IL-1β-NLRP3 inflammasome positive feedback loop by downregulating NF-κB signaling and mtROS production. **a**–**c** Western blot and RT-qPCR analysis of NLRP3 in NP cells cultured with IL-1β, si-P65, si-P65, and melatonin plus IL-1β. **d** IF analysis of NLRP3 in NP cells exposed to si-P65 and si-P65 plus IL-1β (original magnification ×1 000; scale bar = 10 µm). **e**–**g** Western blot and RT-qPCR analysis of SOD2 in NP cells treated with IL-1β, melatonin, and melatonin plus IL-1β. **h**, **i** MitoSOX red staining and quantitative analysis of NP cells cultured with melatonin and melatonin plus IL-1β (original magnification ×1 000; scale bar = 10 µm). CTR, Control; Met, melatonin; Met + IL-1β, melatonin plus IL-1β. *P < 0.05; **P < 0.01; ^#^*P* < 0.05; ^##^*P* < 0.01. The data are shown as the means ± SD

We next assessed the underlying mechanism by which melatonin disrupts the IL-1β-NLRP3 inflammasome positive feedback loop. First, we demonstrated that melatonin significantly suppressed NF-κB signaling activation (Fig. S2). Subsequently, an siRNA targeting P65 (si-P65) was established and verified in NP cells (Fig. S3). Consistently, the NLRP3 level significantly decreased when these cells were transfected with si-P65, and melatonin or si-P65 obviously inhibited the IL-1β-induced increase in NLRP3 expression (Fig. 7a–c). Furthermore, IF staining also revealed that si-P65 attenuated NLRP3 expression in NP cells treated with si-P65 plus IL-1β (Fig. 7d, e). In addition, melatonin prevented the IL-1β-induced decrease in SOD2 expression in NP cells (Fig. 7f–h). MitoSOX Red staining showed that mtROS production was significantly reduced in NP cells of the melatonin plus IL-1β treatment groups (Fig. 7i, j). Therefore, these results confirmed that melatonin disrupted the IL-1β positive feedback loop by suppressing NF-κB signaling and mtROS production in vitro.

### Melatonin disrupts the IL-1β/NF-κB-NLRP3 inflammasome positive feedback loop in vivo

**Fig. 8:**
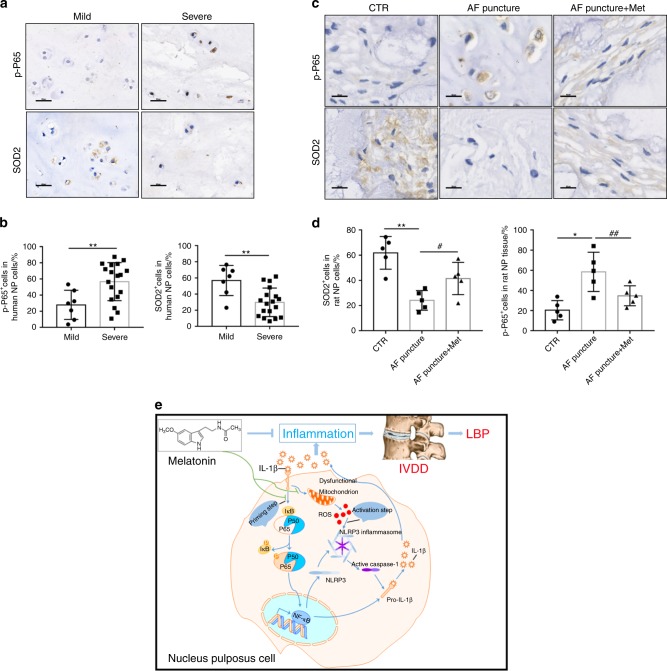
Melatonin disrupts the IL-1β/NF-κB-NLRP3 inflammasome positive feedback loop in vivo. **a**–**d** IHC staining and quantitative analysis of p-P65 and SOD2 in human discs and rats at 8 weeks after the operation (original magnification ×400; scale bar = 50 µm). **e** Proposed schematic representation of melatonin disruption of the IL-1β/NF-κB-NLRP3 inflammasome positive feedback loop to alleviate IVDD in vivo and in vitro. CTR, Control; AF puncture + Met, AF puncture plus melatonin. *n* = 5. *P < 0.05; **P < 0.01; ^#^*P* < 0.05; ^##^*P* < 0.01. The data are shown as the means ± SD

IHC staining showed that, in human samples, p-P65 expression was higher in severely degenerated discs than in mildly degenerated discs, while SOD2 expression was reduced (Fig. 8a, b). Furthermore, the percentage of p-P65-positive cells decreased significantly, while the percentage of SOD2-positive cells increased in melatonin plus AF puncture rats compared with those of AF puncture rats (Fig. 8c, d). These results confirmed that melatonin suppresses the inflammatory response by disrupting the IL-1β/NF-κB-NLRP3 inflammasome positive feedback loop in vitro and in vivo. In conclusion, these results indicated that melatonin disrupts the IL-1β positive feedback loop by suppressing NF-κB signaling and mtROS production and attenuating the progression of IVDD, as illustrated in the proposed schematic representation of melatonin-mediated IVDD in vivo and in vitro (Fig. 8e).

## Discussion

Melatonin is known to have multiple effects, including antioxidant, anti-inflammatory, and antiapoptotic impacts, in different systems.^28,29^ A recent study showed that melatonin downregulates the gene expression of cyclin D1, PCNA, matrix metallopeptidase (MMP)-3, and MMP-9 and upregulates the gene expression of collagen type II alpha 1 chain and aggrecan in NP cells.^47^ Our results were consistent with previous data and showed that the application of melatonin in an IVDD model alleviated the progression of IVDD and LBP. However, whether NLRP3 inflammasome priming and activation are involved in this process is still unknown. Thus, we evaluated the markers of NLRP3 inflammasome priming and activation and found that in the IVDD group, the expression of NLRP3, P20, and IL-1β was elevated, indicating the priming and activation of the NLRP3 inflammasome. Furthermore, LPS, which is a proven NLRP3 inflammasome activator, abrogated the effect of melatonin on IVDD, indicating that melatonin acts by inhibiting NLRP3 inflammasome priming and activation. Data from human disc samples also showed that in the degenerative disc, NLRP3 inflammasome priming and activation marker expression were significantly increased, which gives us more confidence that the NLRP3 inflammasome plays an important role in IVDD.

One of the main causes of IVDD is an abnormal inflammatory response, which is induced by the overexpression of inflammatory cytokines, mainly IL-1β, causing NP cell apoptosis, downregulating matrix gene expression or upregulating the expression of collagen- and aggrecan-cleaving enzymes. For the first time, our study showed that IL-1β promotes its own expression by upregulating NLRP3 inflammasome activation. Furthermore, melatonin, an anti-inflammatory molecule, disrupts the IL-1β positive feedback loop by suppressing the NF-κB signaling pathway and decreasing mtROS production.

First, we found that IL-1β induced NLRP3 inflammasome priming and activation and thus promoted its own overexpression. IL-1β has been widely studied in IVDD due to its catabolic effect. Le Maitre et al. reported that IL-1β is synthesized by native disc cells and that treating human disc cells with IL-1β induces an imbalance between catabolism and anabolism.^27^ These responses represent the changes observed during IVDD. Hence, these findings provide a potential strategy for biological therapy by inhibiting IL-1β to prevent and reverse IVDD. Thus, it is essential to study the expression and regulatory mechanism of IL-1β in IVDD. We first found that in degenerated human NP tissue, the expression of NLRP3, p20, and IL-1β was elevated. The NLRP3 inflammasome has been reported to be activated by LPS or hyperosmotic stress in different systems. Dolunay et al. showed that the inhibition of NLRP3/ASC/pro-caspase-1 inflammasome formation and activity prevents LPS-induced inflammatory hyperalgesia in mice.^54,55^ However, whether IL-1β activates the NLRP3 inflammasome has seldom been studied. In this study, we showed for the first time that in NP cells, IL-1β treatment activated the NLRP3 inflammasome in time- and dose-dependent manners. In addition, IL-1β treatment had an effect similar to that of LPS treatment in upregulating NLRP3 and p20 expression. Interestingly, according to previous studies, the NLRP3 inflammasome is a component of the innate immune system that processes pro-IL-1β into a mature cytokine.^17–19^ Recently, Tang et al. reported that honokiol suppresses activation of the TXNIP-NLRP3 inflammasome in H_2_O_2_-stimulated NP cells, thereby inhibiting the activation of downstream inflammatory mediators such as IL-1β.^56^ Therefore, it is reasonable that IL-1β promotes its own expression through NLRP3 inflammasome activation, forming a positive feedback loop.

Furthermore, we studied the mechanism by which IL-1β induces NLRP3 inflammasome priming and activation. Our previous studies showed that in NP cells, IL-1β activates NF-κB signaling through p65 phosphorylation.^51^ There are some studies concerning NLRP3 inflammasome regulation, and various pathways, including the Smad, NFAT, NF-κB, and MAP kinase pathways, regulate NLRP3 expression.^57,58^ Yu et al. showed that hepatitis B e antigen suppresses LPS-induced NLRP3 inflammasome activation and IL-1β production by repressing NLRP3 and pro-IL-1β expression through inhibition of NF-κB phosphorylation and by repressing caspase-1 activation and IL-1β maturation through inhibition of ROS production.^59^ Budai et al. also reported that LPS induces NLRP3 inflammasome regulation through the NF-κB, p38, JNK, and ERK signaling pathways.^60^ In this study, we found that IL-1β induced NLRP3 expression through NF-κB activation. This conclusion was determined by the application of SM7368, a specific NF-κB pathway inhibitor. mtROS are the main activators of the NLRP3 inflammasome;^52,53^ thus, it is important to know whether IL-1β regulates mtROS production. According to our MitoSOX Red staining and western blotting results, IL-1β treatment reduced SOD2 expression and induced mtROS production in NP cells. In conclusion, our results indicated that in NP cells, IL-1β activates the NLRP3 inflammasome through NF-κB signaling activation and mtROS production.

Furthermore, we found that melatonin disrupted the IL-1β positive feedback loop and studied the mechanism of this disruption. Melatonin has also been reported to suppress NLRP3 inflammasome activation and IL-1β expression. Dong et al. showed that melatonin attenuates NLRP3, ASC, cleaved caspase-1, IL-1β, and IL-6 expression.^44^ Consistent with previous reports, our data showed that melatonin suppressed NF-κB signaling activation and reduced mtROS production to inhibit NLRP3 inflammasome activation and IL-1β expression. We confirmed these results by applying p65 siRNA and MCC950 (a specific inhibitor of NLRP3 inflammasome activation). Furthermore, we confirmed our discovery in vivo. Our data showed that in this IVDD model, p-p65 expression was decreased and SOD2 expression was increased after melatonin treatment, indicating that melatonin exerted anti-inflammatory effects via the NF-κB-NLRP3 inflammasome-IL-1β axis.

There were several limitations to this study. First, the number of human IVD tissue samples was relatively small due to the difficulty associated with acquiring grade I/II discs in clinical practice. Second, the detailed mechanisms by which melatonin suppresses NF-κB signaling and mtROS production were not addressed and might be elucidated in future studies.

In conclusion, we found for the first time that IL-1β forms a positive feedback loop through NLRP3 inflammasome activation in IVDD and that melatonin disrupts this vicious cycle by suppressing NF-κB signaling activation and mtROS production. Our research showed a new mechanism by which IL-1β promotes the inflammatory response in IVDD, and melatonin may be used as a therapeutic agent for the treatment of inflammatory cytokine-related IVDD.

## Materials and methods

### Collection of human IVDs

Before tissue collection, each patient signed an informed consent form, and the Medical Ethics Committee of The First Affiliated Hospital of Sun Yat-sen University (no: [2017] 203) approved this study. All studies in this paper were performed according to The Code of Ethics of the World Medical Association (Declaration of Helsinki).^61^ Between March 2016 and April 2018, we collected 25 disc samples (detailed information about the specimens is in Table 1) from patients (male: female, 13: 12). The degree of disc degeneration was evaluated by Pfirrmann classification. Normal discs were obtained from patients with trauma and deformity, and degenerated discs were obtained from patients with degenerative spinal diseases (disc herniation, spinal canal stenosis, and degenerative scoliosis).

**Table 1:** Information of human disc samples from 25 patients

Human disc samples	Sex	Age	Diagnosis	Level	Grade
1	M	17 y	AIS	T9/10	I
2	F	14 y	AIS	T7/8	I
3	M	16 y	AIS	T11/12	I
4	F	15 y	AIS	T11/12	II
5	M	18 y	Trauma	L1/2	II
6	F	19 y	Trauma	L3/4	II
7	F	23 y	Disc herniation	L3/4	II
8	F	31 y	Disc herniation	L4/5	III
9	M	29 y	Disc herniation	L2/3	III
10	M	34 y	Disc herniation	L3/4	III
11	F	42 y	Disc herniation	L4/5	III
12	F	36 y	Disc herniation	L4/5	III
13	M	27 y	Disc herniation	L4/5	III
14	M	30 y	Disc herniation	L2/3	IV
15	M	39 y	Disc herniation	L3/4	IV
16	M	47 y	Disc herniation	L2/3	IV
17	F	58 y	Disc herniation	L3/4	IV
18	F	67 y	Disc herniation	L3/4	IV
19	M	59 y	Disc herniation	L4/5	IV
20	F	69 y	Disc herniation	L3/4	V
21	M	63 y	Disc herniation	L4/5	V
22	M	83 y	Disc herniation	L3/4	V
23	F	68 y	Disc herniation	L3/4	V
24	M	74 y	Disc herniation	L2/3	V
25	F	78 y	Disc herniation	L3/4	V

*AIS* adolescent idiopathic scoliosis, *M* male, *F* female, *y* years

### Cell culture and cell viability assay

As previously described,^62^ NP cells were isolated and cultured in DMEM (Invitrogen, CA, USA) containing 10% fetal bovine serum (Invitrogen, CA) and penicillin/streptomycin (Invitrogen, CA) at 37 °C in a humidified incubator with 5% CO_2_. We harvested NP cells using solutions containing trypsin (0.25%) and EDTA (1 mM) (Invitrogen, CA) and subcultured the cells in 10 cm dishes. NP cells were seeded in six-well plates, grown to ~80% confluence and treated with melatonin (1 mmol·L^−1^, M5250, Sigma-Aldrich, USA), MCC950 (10 nmol·L^−1^, Selleck, a specific inhibitor of NLRP3 inflammasome activation), lipopolysaccharide (LPS, 200 ng·mL^−1^, L2880, Sigma-Aldrich, USA), TNF-α (100 ng·mL^−1^, 210-TA-020, R&D Systems, USA), or IL-1β (50 ng·mL^−1^, 201-LB-005, R&D Systems, USA) for 24 h for subsequent experiments. The cytotoxic effect of melatonin on NP cells was detected using a cell counting kit (CCK)-8 assay (Dojindo Laboratories, Kumamoto, Japan) according to the manufacturer’s instructions.

### Small interfering RNA (siRNA) transfection

Rat NP cells were seeded at 5 × 10^6^ per well in a six-well plate and transfected with negative control or siRNA targeting P65 (RiboBio, Guangzhou, Guangdong, China) when the cells reached 60%–70% confluence. The sequences for the P65-specific siRNAs were as follows: 1: GCTGCAGTTTGATGATGAA, 2: GCCCTATCCCTTTACGTCA, and 3: GGACATATGAGACCTTCAA (10 nmol·L^−1^). Then, we used 250 μL of serum-free optical-MEM (Invitrogen, CA, USA) to individually dissolve 5 µL of siRNA or 10 μL of Lipofectamine 3000 (Invitrogen, CA, USA). After mixing them together, the mixture was added to the cells. After treatment, the cells were harvested for protein/RNA extraction.

### Western blot analysis

The proteins of treated NP cells were extracted and electrophoretically separated via 10% or 15% SDS-PAGE, as previously described.^63^ Subsequently, the membranes were blocked with 3% bovine serum albumin and incubated with primary antibodies. The primary antibodies included anti-pro-IL-1β (1:1 000, 12703, Cell Signaling Technology), anti-IL-1β (1:1 000, ab8320, Abcam), anti-phospho-P65 (1:1 000, 3033, Cell Signaling Technology), anti-P65 (1:1 000, 8242, Cell Signaling Technology), anti-phospho-Erk1/2 (1:1 000, 4370, Cell Signaling Technology), anti-Erk1/2 (1:1 000, 4695, Cell Signaling Technology), anti-phospho-P38 (1:1 000, 4511, Cell Signaling Technology), anti-P38 (1:1 000, 8690, Cell Signaling Technology), anti-NLRP3 (1:1 000, AG-20B-0014, AdipoGen), anti-cleaved Caspase-1 (p20) (1:1 000, AG-20B-0042, AdipoGen), anti-ASC (1:1 000, AG-25B-0006-C100, AdipoGen), anti-Caspase-1 (1:1 000, ab1872, Abcam), anti-superoxide dismutase 2 (SOD2) (1:1 000, 13141, Cell Signaling Technology), and anti-β-actin (1:3 000, 4970, Cell Signaling Technology). After washing with PBS, the membranes were incubated with the following secondary antibodies: anti-rabbit IgG (1:5 000, 7074, Cell Signaling Technology) or anti-mouse IgG (1:5 000, 7076, Cell Signaling Technology). Finally, the Western blot bands were detected using enhanced chemiluminescence detection reagents (Invitrogen, CA, USA) and quantified using ImageJ software (National Institutes of Health, Bethesda, MD, USA).

### Real-time quantitative polymerase chain reaction (RT-qPCR)

The isolation of total RNA from NP cells was performed using RNAiso Plus (Takara, Japan). Complementary DNA synthesis was performed using a Prime Script RT Master Mix kit (Takara) according to the manufacturer’s instructions. SYBR green Premix Ex Taq II (Takara) was used to detect the relative mRNA levels of NLRP3, pro-IL-1β, SOD2, P65, and β-Actin, and the sequences of the primer pairs are listed in Table 2 (Sangon Biotech, Shanghai, China). RT-qPCR was performed on an ABI 7900HT Fast Real-Time PCR System (Applied Biosystems) for 40 cycles and quantified using the 2^−ΔΔCt^ method.

**Table 2:** Specific primers

Primers	sequences
NLRP3 R	5′- ATCAACAGGCGAGACCTCTG-3′
NLRP3 F	5′- ATCAACAGGCGAGACCTCTG-3′
SOD2 R	5′- GCGTTGATGTGAGGTTCCAG-3′
SOD2 F	5′- GCTCCGGTTTTGGGGTATCTG-3′
P65 R	5′- GTTCACGGATGACCTCTTTGTTT-3′
P65 F	5′- GGGCTTGGAAATAGAGACATTGA-3′
IL-1β R	5′-CACACACTAGCAGGTCGTCA -3′
IL-1β F	5′-CCTATGTCTTGCCCGTGGAG -3′
β-Actin R	5′-GGATGGCTACGTACATGGCTG -3′
β-Actin F	5′-CATTGTCACCAACTGGGACGATA -3′

### MitoSOX Red and immunofluorescence (IF) staining

NP cells were treated as described, incubated with 2.0 mL of 5 μmol·L^−1^ MitoSOX Red reagent or fixed with 4% paraformaldehyde and then blocked with 5% normal goat serum. Subsequently, the cells were incubated with anti-NLRP3 (1:200, ab4207, Abcam) and anti-phospho-P65 (1:200, 3033, Cell Signaling Technology) antibodies overnight, followed by incubation with Alexa Fluor-488-conjugated anti-rabbit and anti-goat secondary antibodies (Invitrogen, 1:2 000). Nuclear staining was performed using DAPI. Finally, the cells were photographed under an Olympus BX63 microscope (Tokyo, Japan) at ×400 and ×1 000 magnifications.

### Animal model and magnetic resonance imaging (MRI) evaluation

As previously described,^64^ the rats (weighing 200–250 g, *n* = 5 per group) were divided into three groups: the blank group received no puncture and was intraperitoneally injected with 0.9% normal saline; the other two groups underwent AF puncture surgery with a 21-gauge needle inserted 3.0 mm into the L3/4 IVDs for 30 s. At 4 or 8 weeks post operation, MRI (T2-weighted images) signal change examinations were performed on the rats (*n* = 5 per group). In addition, the degree of disc degeneration was evaluated by Pfirrmann classification.

Melatonin was diluted to 20 mg·mL^−1^ with normal saline and then intraperitoneally injected into the rats (30 mg·kg^−1^ per week), in addition to LPS (2 mg·kg^−1^ per week), until the day of euthanasia.

### Immunohistochemistry (IHC) and histopathological analysis

The specimens were embedded in paraffin and cut into 5 µm sections. Subsequently, the sections were deparaffinized and rehydrated, followed by H&E and Safranin-O staining or antigen retrieval with 0.01 mol·L^−1^ sodium citrate. The sections were blocked with 3% hydrogen peroxide and 5% normal goat serum. Then, the slides were incubated with the following primary antibodies: anti-NLRP3 (1:200, AG-20B-0014, AdipoGen), anti-cleaved caspase-1 (p20) (1:200, AG-20B-0042, AdipoGen), anti-IL-1β (1:200, ab8320, Abcam), anti-SOD2 (1:1 000, 13141, Cell Signaling Technology), and anti-phospho-P65 (1:200, 3033, Cell Signaling Technology). The sections were incubated with a secondary antibody and then developed with DAB solution. Hematoxylin was used for nuclear staining. Finally, the sections were observed and imaged under an Olympus BX63 microscope at ×10, ×50, and ×400 magnifications, and the percentages of NLRP3^+^, IL-1β^+^, p20^+^, SOD2^+^, and phospho-P65^+^ cells in the IVD samples were quantified using ImageJ software (National Institutes of Health, Bethesda, MD, USA).^65^ The histologic scores were assessed as previously described: normal disc, five; moderately degenerated disc, 6–11; and severely degenerated disc, 12–14.^66,67^

### Behavioral study

The rats were subjected to behavioral tests as described in our previous study.^63^ Reflex reactions to both mechanical and harmful thermal stimuli applied to both hind paws were measured in five rats from each group within a week of surgery and every 2 weeks during the following 10 weeks.

### Statistical analysis

The differences among the groups were assessed by one-way analysis of variance (ANOVA), which was performed using SPSS software version 19.0 for Windows (IL, USA). If the ANOVA results were statistically significant, the differences between the two groups were examined by using Bonferroni’s post hoc test. The data are presented as the mean ± SD. *P* values < 0.05 were considered statistically significant.

## Supplementary information

Figure S1

Figure S2

Figure S3

Supplementary figure legends
