# Supplementary figures and images for: Melatonin alleviates intervertebral disc degeneration by disrupting the IL-1β/NF-κB-NLRP3 inflammasome positive feedback loop
Source: Bone Res. 2020 Feb 18;8:10. doi: 10.1038/s41413-020-0087-2 (PMC7028926; doi:10.1038/s41413-020-0087-2)

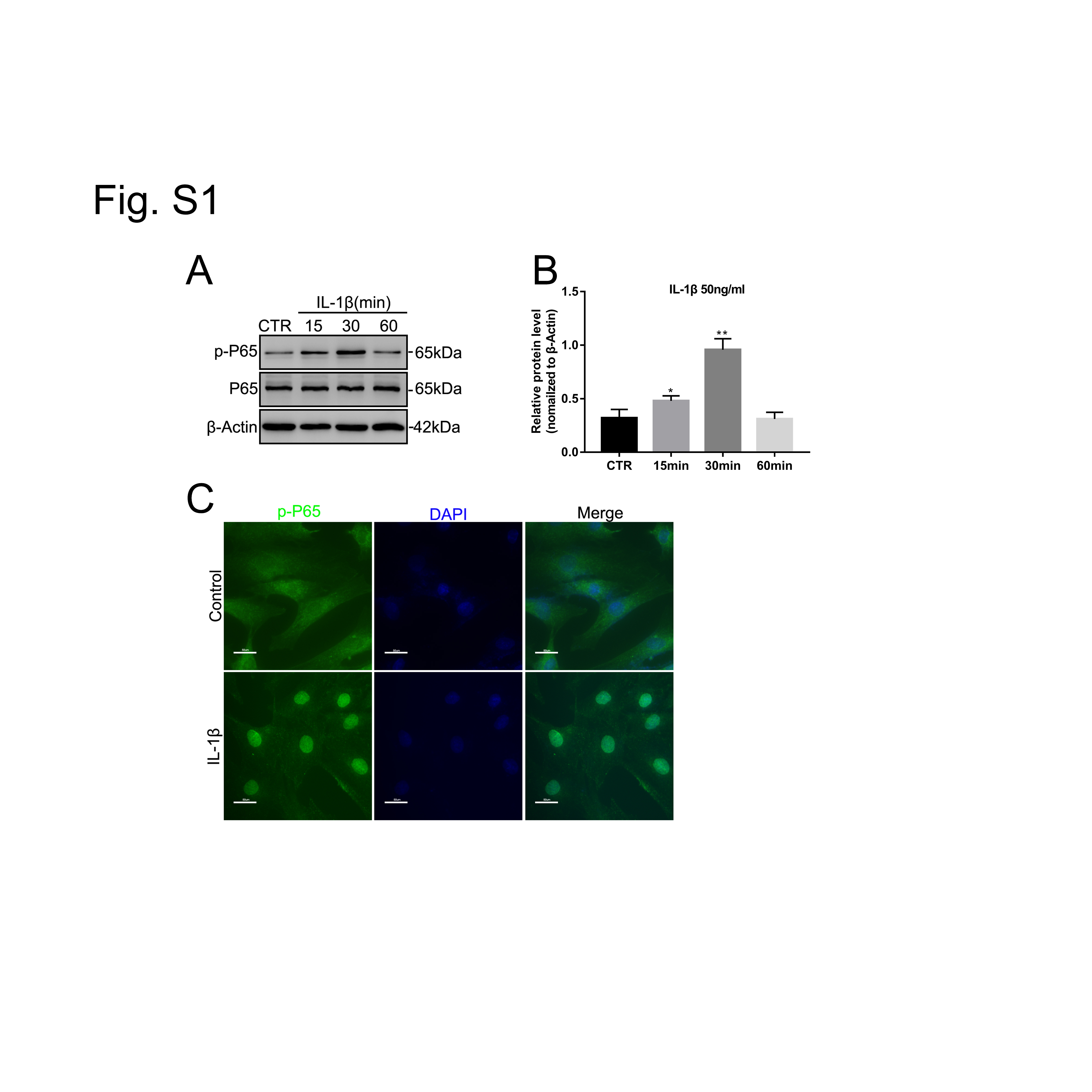

Supplement: Supplementary file 1 — Figure S1 [file 41413_2020_87_MOESM1_ESM.jpg]

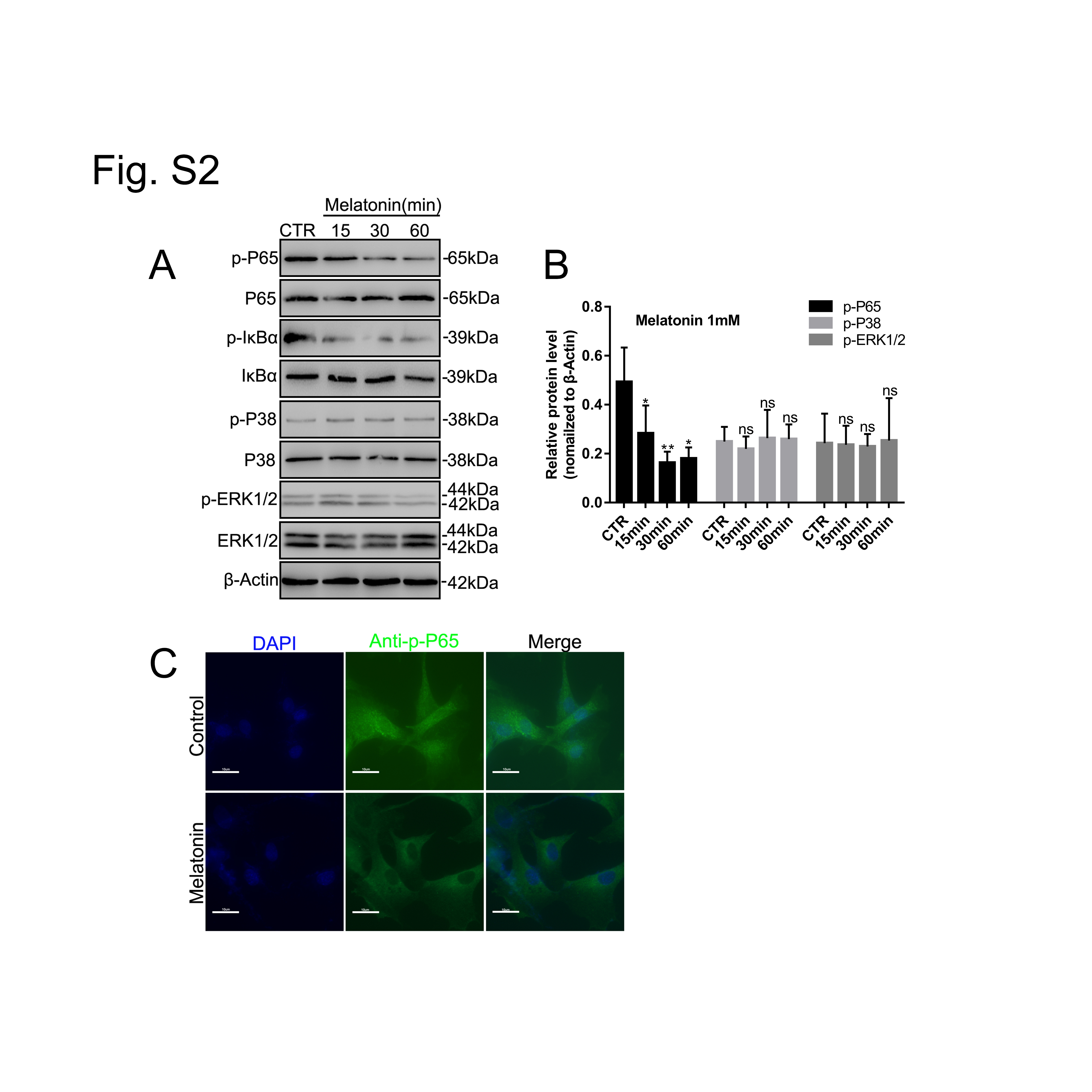

Supplement: Supplementary file 2 — Figure S2 [file 41413_2020_87_MOESM2_ESM.jpg]

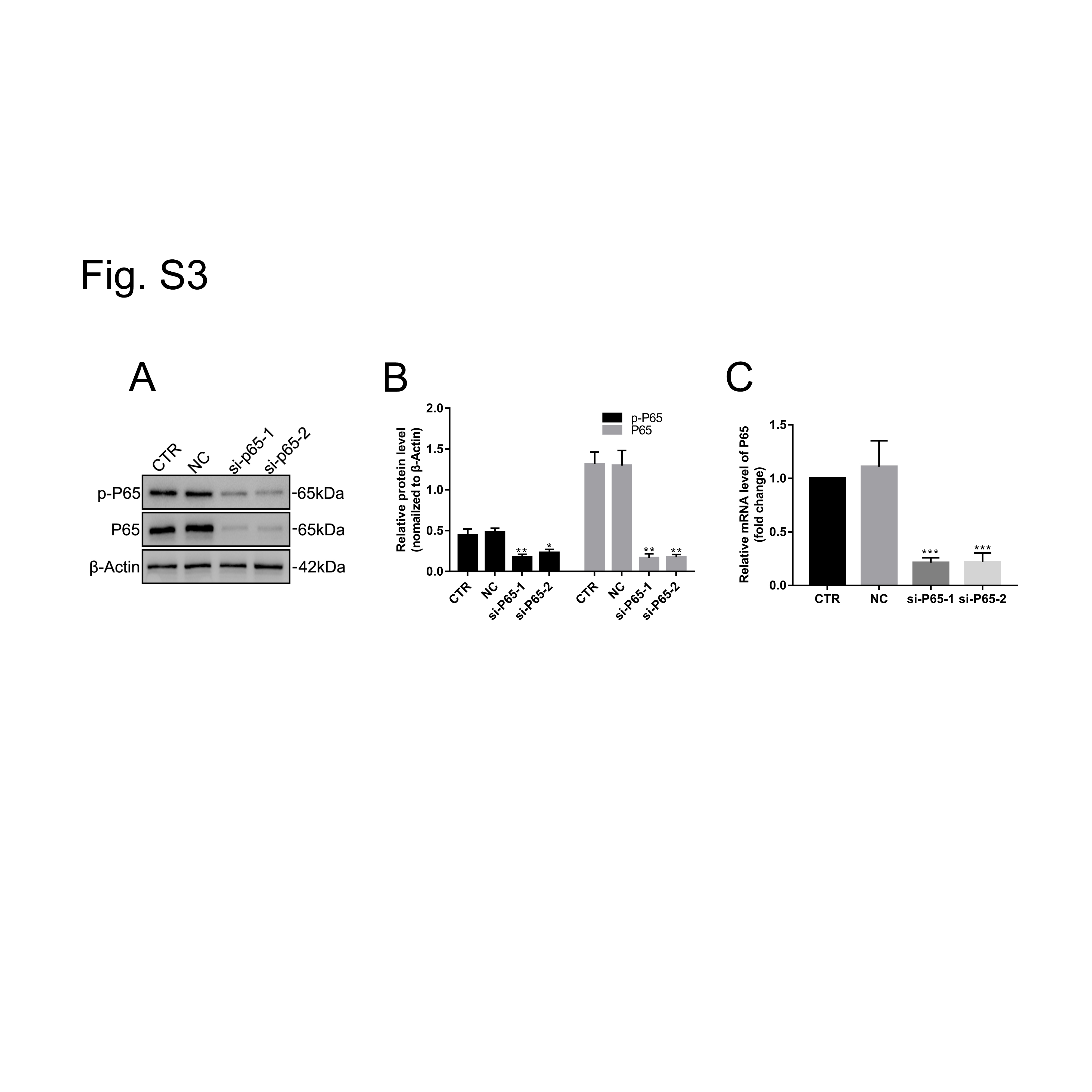

Supplement: Supplementary file 3 — Figure S3 [file 41413_2020_87_MOESM3_ESM.jpg]
