# Supplementary material for: Melatonin alleviates intervertebral disc degeneration by disrupting the IL-1β/NF-κB-NLRP3 inflammasome positive feedback loop
Source: Bone Res. 2020 Feb 18;8:10. doi: 10.1038/s41413-020-0087-2 (PMC7028926; doi:10.1038/s41413-020-0087-2)
Supplement: Supplementary file 4 — Supplementary figure legends [file 41413_2020_87_MOESM4_ESM.docx]

**Figure S1: IL-1β activates NF-κB signaling in NP cells. (A and B)** Western blot showed that the NF-κB signaling was obviously activated in NP cells treated with IL-1β (20 ng/ml, 30 min). **(C)** IF staining also showed that IL-1β induced NF-κB signaling activation in NP cells. CTR=Control; *p < 0.05, **p < 0.01. Data are shown as the means ± SD.

**Figure S2: melatonin suppresses NF-κB signaling activation in NP cells. (A and B)** Western blot showed that a significant decrease in NF-κB signaling was detected in NP cells treated with melatonin (1 mM, 30 min) by Western blot analysis, but no changes were observed in the P38 MAPK and Erk1/2 signaling pathways under the same treatment. **(C)** IF staining showed that melatonin significantly inhibited NF-κB signaling activation in NP cells. CTR=Control; ns= no statistical significance, *p < 0.05, **p < 0.01. Data are shown as the means ± SD.

**Figure S3: P65 level is decreased in NP cells transfected with si-P65. (A and B)** Western blot showed that si-P65 significantly reduced the P65 expression in NP cells. **(C)** RT-qPCR analysis showed that P65 level downregulated in NP cells transfected with si-P65. CTR=Control; *p < 0.05, **p < 0.01, ***p < 0.001. Data are shown as the means ± SD.
